# Harnessing peptide hormones for postharvest preservation of horticultural produce

**DOI:** 10.3389/fpls.2025.1700360

**Published:** 2025-10-15

**Authors:** Junpeng Niu, Huizhen Bao, Sibo Jiao, Huibin Han, Guodong Wang

**Affiliations:** ^1^ College of Life Sciences, Engineering Research Center of High-Valued Utilization of Fruit Resources in Western China of Ministry of Education, Key Laboratory of Medicinal Resources and Natural Pharmaceutical Chemistry of Ministry of Education, Shaanxi Normal University, Xi’an, China; ^2^ College of Bioscience and Bioengineering, Jiangxi Province Key Laboratory of Vegetable Cultivation and Utilization, Jiangxi Agricultural University, Nanchang, China

**Keywords:** horticultural produce, peptide hormone, postharvest preservation, shelf life, small peptide

## Introduction

Horticultural postharvest losses, including reductions in quantity, quality and appearance from harvest to consumption, pose a significant global challenge to the supply chain and economic sustainability. Postharvest losses occur more frequently in fruits and vegetables due to their high moisture content, bulkiness, and high respiratory rate as living tissues (Ali et al., 2025). Furthermore, the seasonality, regionality, and perishability of fresh fruits and vegetables substantially complicate postharvest storage and shelf life, ultimately affecting consumer acceptance. Accumulating studies focused on addressing postharvest losses in horticultural produce, underscoring the importance and essential role of innovative preservation technologies to mitigate this challenge. To date, a plethora of methods including physical preservation (e.g., cold storage, modified atmosphere storage, edible coatings, heat treatment, and irradiation), chemical preservation (e.g., 1-methylcyclopropene (1-MCP), calcium chloride, hydrogen sulfide, diphenylamine, and synthetic compounds), and biological preservation (e.g., antibiotics and bacterial inhibitors) have been shown to effectively prolong the supply period and maintain the nutritional quality of fruits and vegetables (Sridhar et al., 2021; You et al., 2022; Gomes et al., 2023). However, conventional preservation methods present significant environmental and safety concerns. Physical refrigeration, for instance, carries a high environmental cost due to its substantial energy consumption and carbon emissions. Chemical preservatives such as diphenylamine not only pose environmental pollution risks but also cause adverse health effects including skin hypersensitivity, tissue dysfunction, impaired endocrine function, and cancer (Ahmed et al., 2022). While effective, 1-MCP can impair fruit quality like flavor, aroma, and color, and may even induce abnormal texture or trigger physiological diseases in certain horticultural products (Ahmed et al., 2022). Furthermore, current biological preservation methods are often hampered by unstable efficacy, high development cost, and complex application technologies. Consequently, the development of more efficient, stable, cost-effective, and environment-friendly strategies are imperative to mitigate potential these adverse impacts on human health and the environment.

Small peptides are short-chain biological molecules, typically consisting of 2–100 amino acids, that are generally classified into conventional peptides and non-conventional peptides (Krouk et al., 2023; Ji et al., 2025; Zhang et al., 2025). Conventional peptides are derived from well-characterized coding regions or standard open reading frames (ORFs), consisting of post-translationally modified peptides, cysteine-rich peptides, and non-cysteine-rich/non-post-translationally modified peptides (Ji et al., 2025; Zhang et al., 2025). Peptide hormones predominantly comprise post-translationally modified peptides and cysteine-rich peptides. In contrast, non-conventional peptides, such as micropeptides and small ORF-encoded polypeptides, originate from noncoding regions, including untranslated sequences, introns, non-coding RNAs (pri-miRNAs, long non-coding RNAs, and circular RNAs), intergenic regions, and other non-coding sequences (Zhang et al., 2025). As critical regulators with multifaceted roles, small peptides function in diverse fields such as medicine, cosmetics, food preservation, animal nutrition and healthcare, and plant growth and defense (Fan et al., 2022; Krouk et al., 2023; Zhang et al., 2023; Ji et al., 2025; Zhang et al., 2025). Their versatile functionality is attributable to a combination of favorable properties, including abundant sources, structure diversity, high specificity, membrane permeability, biodegradability, low toxicity, and complex receptor interactions (Fan et al., 2022; Zhang et al., 2023, 2025).

Currently, small peptides used in fruit ripening and postharvest management predominantly are non-conventional peptides, including antimicrobial peptides, antioxidative peptides, and peptides that providing physical barrier and/or regulating protein-protein interactions (Fan et al., 2022; Zhang et al., 2023). For instance, NOP-1, a small non-conventional peptide derived from the C-terminal part of the Arabidopsis ethylene regulator ETHYLENE INSENSITIVE2 (EIN2), binds to the ETHYLENE RESPONSE1 (ETR1) receptor. The binding prevents the formation of the EIN2-ETR1 protein complex in tomato, thereby blocking ethylene signaling and delaying the ripening process when applied to tomato (*Solanum pimpinellifolium* L.) fruit surface; furthermore, a significant delay in maturation and reddening is observed for exogenous applications of NOP-1 via injection, incubation, or surface application (Bisson et al., 2016; Milić et al., 2018). However, unlike conventional peptides, the non-conventional peptides are derived from highly divergent biosynthesis and processing routes which heavily hinders their identification, functional studies, and exploration of their molecular mode-of-action. In this regard, the classic type of conventional peptides, especially the peptide hormones with clear functions and well-defined molecular mechanisms, represent potential candidates to develop novel preservatives for extending storage and shelf life of horticultural products.

## Peptide hormones are critical regulators in plant plasticity under abiotic and biotic stress

Plant peptide hormones are the classic type of conventional peptides which mediate cell-to-cell communications across diverse biological processes (Ji et al., 2025; Zhang et al., 2025). Typically, mature peptide hormones are undergone proteolytic processing and post-translational modifications from prepropeptides, which commonly harbor an N-terminal signal peptide for secretion, a variable intermediate sequence, and a conserved functional domain (Zhang et al., 2025). Subsequently, matured peptide hormones are secreted into the apoplast, where they diffuse to target cells over several cell layers or travel a long distance via xylem and phloem (Zhang et al., 2025). Commonly, peptide hormones are perceived by the membrane-localized receptor(s) to (de)activate either long-distance or local signaling pathways, thereby orchestrating plant developmental plasticity and stress resilience via (post)transcriptional, (post)translational, and/or epigenetic regulations (Ji et al., 2025; Zhang et al., 2025). Extensive studies have shown that peptide hormones are comprehensively implicated in, but not limited to, diverse biological processes such like stem cell homeostasis, organogenesis, fertilization, senescence, abiotic stress responses, and plant-pathogen interactions (Ji et al., 2025; Zhang et al., 2025). Particularly, peptide hormones are deeply implicated in the signaling and/or biosynthesis of phytohormones such as ethylene (Wang et al., 2016; Zhang et al., 2025), which is the gaseous ripening hormone notably influencing the postharvest physiology of the fruit. Importantly, many peptide hormones are able to confer tolerance to senescence, cold stress, and pathogen diseases which are deleterious during postharvest storage. Although significant advances have been achieved in understanding peptide hormones, the translation of this knowledge into practical applications remains limited. In this regard, peptide hormones with defined roles in crosstalking with ethylene or conferring tolerance to senescence, cold stress, and pathogen diseases are promising candidates for postharvest longevity.

## Peptide hormones in preservation of postharvest horticultural products

**Figure 1:**
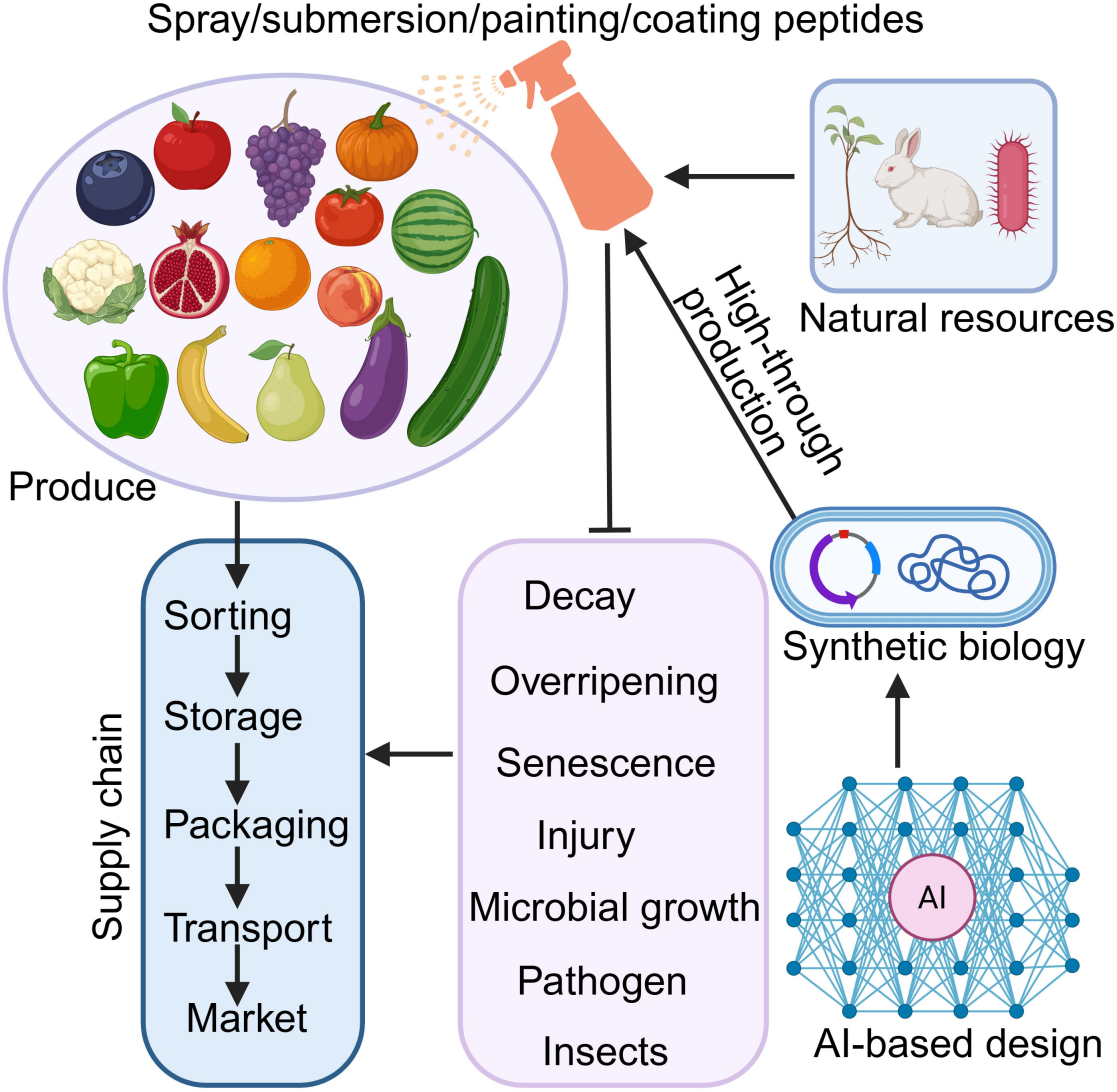
A simplified model of peptide hormones in the postharvest preservation and quality control of horticultural produce. Application of peptide hormones, either naturally sourced or chemically synthesized via artificial intelligence and synthetic biology, can exert inhibitory effects on overripening, decay, senescence, physical damage, microbial growth, pathogen invasion, and pest infestation, thereby extending postharvest longevity and sustaining nutritional quality of horticultural products.

Growing evidence have now surfaced that small signaling peptides can be used to delay fruit ripening and senescence and deterioration of horticultural produce, thereby reducing postharvest losses and maintaining commercial value (**Figure 1**; **Table 1**). The CTG134 peptide, a ROOT GROWTH FACTOR/GOLVEN (RGF/GLV) peptide that is transcriptionally activated by 1-MCP, is essential for maintaining the balance between auxin and ethylene during peach (*Prunus persica* L. Batsch) ripening and thus extending peach fruit storage duration (Tadiello et al., 2016). It was further shown that there are significant differences in peptidome between melting-flesh (‘Zhongyoutao No.13’) and stony-hard peach (‘Zhongyoutao No.16’) cultivars during ripening. Those differentially abundant peptides are found to be predominantly involved in starch and sucrose metabolism, glycolysis, and ribosome synthesis, suggesting that these metabolic pathways are involved in peach fruit ripening and providing a theoretical reference for further exploring of RGF/GLV peptides in regulating peach fruit ripening and senescence (Li et al., 2022). Exogenous application of another peptide hormone, PHYTOSULFOKINE (PSK), can effectively retard yellowing and preserve the nutritional integrity of broccoli (*Brassica oleracea* L.var. *italica* Plenck) florets under cold storage through elevated levels of guanosine 3′, 5′-cyclic monophosphate (cGMP), phenols, flavonoids, and ascorbic acid (Aghdama et al., 2020). In addition, exogenous PSK application has been also shown to delay yellowing in broccoli florets by increased activities of methionine sulfoxide reductase, antioxidants, cysteine peroxiredoxin, and enhanced hydrogen sulfide production, alongside upregulating the expression levels of genes encoding SUMO E3 ligase SIZ1 and HEAT SHOCK PROTEINs (HSPs) 70/90 (Aghdam and Flores, 2021; Aghdam et al., 2021a). On the other side, PSK peptides decrease the activities of phospholipase D and lipoxygenase, thus reducing endogenous hydrogen peroxide (H_2_O_2_) accumulation and protecting membrane integrity featured as lower malondialdehyde accumulation, all of which collectively delays senescence in broccoli florets (Aghdam and Flores, 2021). Similarly, PSK application delays petal senescence in cut rose (*Rosa hybrida* cv. ‘Angelina’) flowers by potentiating SUMO1/SUMO E3 ligase SIZ1 signaling and suppressing endogenous H_2_O_2_ accumulation (Aghdam et al., 2021b). In litchi (*Litchi chinensis* Sonn. cv. ‘Guiwei’), PSK peptide application has also been shown to effectively impede litchi pericarp browning by modulating oxidative enzymatic reactions and suppressing the expression of browning-related genes (Liang et al., 2025). A recent genetic and molecular study demonstrated that the exogenous application of sulfated, mature PSK peptide facilitates fruit color transition, ripening, and quality establishment of tomato (*Solanum lycopersicum* L. cv. ‘Condine Red’) fruits by inducing phosphorylation of the transcription factor DEHYDRATION-RESPONSIVE ELEMENT BINDING PROTEIN 2F (DREB2F) via the PSK RECEPTOR 1 (PSKR1) receptor (Fang et al., 2024). Importantly, peptide hormones exhibit high binding affinities, high selectivity and specificity, and good efficacy in their actions (Ji et al., 2025; Zhang et al., 2025), thereby avoiding non-specific, side, or off-target adverse effects. High functional specificity and binding affinity of peptide hormones also contribute to their favorable safety profile and low frequency of off-target effects. Their general hydrophilicity further reduces the potential for tissue accumulation. Furthermore, peptide hormones are typically degraded into non-toxic metabolites (amino acids). Moreover, since peptide hormones derived from plant sources offer an environmentally safe way for regulating fruit and vegetable production without causing environmental pollution. Taken together, these studies demonstrate that, acting as function-specific, good efficacy, safer, and environmentally friendly alternatives, peptide hormones play curial roles in delaying senescence, extending shelf life, and maintaining fruit quality during postharvest preservation (**Table 1**).

**Table 1:** A summary of peptide hormones in postharvest preservation.

Peptides	Plant species	Function	Downstream signaling	Reference
RGFGLV (CTG134 peptide)	Peach	Ripening	Auxin and ethylene	Tadiello et al., 2016
PSK	Peach	Cold tolerance	ROS and cell wall metabolisms	Zeng et al., 2024
PSK	Broccoli	Delay yellowing and preserve the nutritional integrity under cold	cGMP, phenols, flavonoids, and ascorbic acid	Aghdama et al., 2020
PSK	Broccoli	delays senescence	Phospholipase D, lipoxygenase, H_2_O_2_	Aghdam and Flores, 2021
PSK	Rose	Delay petal senescence	H_2_O_2_	Aghdam et al., 2021b
PSK	Tomato	Fruit ripening	DREB2F	Fang et al., 2024
PSK	Loquat	Chilling tolerance	Energy status and cell integrity	Song et al., 2017
PSK	Bananas	Chilling injury	Nitric oxide, polyamine, proline, and γ-Aminobutyric	Wang et al., 2022
PSK	Strawberry	Relieve fungal decay, delay senescence	ROS, ATP	Aghdam and Alikhani-Koupaei, 2021; Aghdam et al., 2021c, 2021
RALF33	Strawberry	Anthracnose ontogenic resistance		Merino et al., 2019

Peptide hormones are also known to play roles in response to abiotic stress and pathogenic invasions (Wang et al., 2016; Ji et al., 2025; Zhang et al., 2025), underscoring their potential to alleviate adverse conditions and infectious diseases in fruits and vegetables during postharvest preservation (**Figure 1**). In loquat (*Eriobotrya japonica* (Thunb.) Lindl. cv. ‘Luoyangqing’) fruits, a cold-induced PSK peptide PSK1 was found to improve chilling tolerance of loquat fruit by sustaining high energy status and cell integrity, exhibiting retardation of internal browning development and weight loss in PSK1-treated fruits (Song et al., 2017). Furthermore, exogenous application of PSK peptides also confer tolerance to chilling injury, thus repressing internal browning, delaying senescence, and preserving nutritional quality of loquat (*Eriobotrya japonica* Lindl. cv. ‘Jiefangzhong’) fruits during cold storage (Liu et al., 2024). Mechanistically, PSK peptide alleviates chilling injury and maintains fruit quality during of loquat during cold storage, which is achieved by regulating the metabolism of sugar, proline, polyamines, and γ-aminobutyric acid (Liu et al., 2024). In bananas (*Musa acuminata* cv. ‘Brazil’), PSK application similarly mitigates chilling injury, such as cold-induced browning and water-soaking by regulating nitric oxide, polyamine, proline, and γ-aminobutyric acid metabolisms (Wang et al., 2022). PSK treatment also can delay and alleviate the flesh browning caused by chilling injury, thereby preserving quality and flavor of postharvest peach (*Prunus persica* (L.) Batsch. cv. ‘Hujingmilu’) fruit via modulating ROS and cell wall metabolisms (Zeng et al., 2024). Beyond abiotic stress, exogenously applied of PSK peptides have been shown to relieve fungal decay and delay senescence in strawberry (*Fragaria* × *anannasa* cv. ‘Selva’ and ‘Camarosa’) fruits through activating extracellular ATP signaling, improving REACTIVE OXYGEN SPECIES (ROS) scavenging, and promoting antioxidant nutrient accumulation during cold storage (Aghdam and Alikhani-Koupaei, 2021; Aghdam et al., 2021c, 2021). In addition, the RAPID ALKALINIZATION FACTOR 33 (RALF33) peptide has been shown to negatively regulate anthracnose ontogenic resistance in unripe strawberry (*Fragaria* × *ananassa* cv. ‘Alba’) fruits (Merino et al., 2019). Altogether, these findings demonstrate that small signaling peptides are able to combat both abiotic and biotic stresses during postharvest preservation, leading to prolonged storage duration and improved quality of fruits and vegetables (**Table 1**). Given that many peptide hormones are heavily implicated in stress acclimatization (Wang et al., 2016; Ji et al., 2025; Zhang et al., 2025), it is expected that additional peptide hormones could be assigned with roles in postharvest preservation of fruits and vegetables.

## Future challenges and perspectives

Despite their considerable potential, the full utilization of small signaling peptides in postharvest preservation is constrained by several limitations, including poor stability, limited bioavailability, inefficient delivery into target tissues, and high production costs. Consequently, future peptide production must prioritize high stability, low cost, and safety. Artificial Intelligence (AI) and synthetic biology represent promising methodologies for next-generation peptide production, enabling enhance stability, optimize formulations, and enable cost-effective, high-throughput manufacturing (**Figure 1**). For instance, the AI-based design of antiproteolysis peptide, APP3-14, has been demonstrated to control Huanglongbing, a catastrophic disease affecting citrus crops (Zhao et al., 2025). By integrating deep learning algorithms, Zhao et al. (2025) efficiently identified a series of therapeutic peptides, including APP3–14 which significantly inhibits the colonization of the Huanglongbing pathogen and disrupt its transmission cycle. In this context, a couple of successful AI platforms and tools, for instance, AlphaFold-Multimer, neural language models (NLMs), variational autoencoders (VAEs), generative adversarial networks (GANs), and the PepINVENT tool can be applied to design novel small peptides for horticultural produce preservation (Wan et al., 2022; Geylan et al., 2025). As such, the AI-aided improved stability, low toxicity, and enhanced functional specificity of peptide hormones offer environment-friendly alternatives for postharvest longevity.

Over 30 families of peptide hormones were identified across plant species (Zhang et al., 2025), whereas only a few have been characterized for postharvest preservation roles. Given that many peptide hormones are involved in response to senescence, chilling stress, and pathogen diseases that are frequently occur during postharvest storage, further exploration is expected to reveal additional peptide hormones with functions in postharvest management of fruits and vegetables. Preharvest interventions are equally critical; thus, it is very likely that applying peptide hormones could preempt latent infections or reinforce prepared cellular integrity. Peptide hormones can be exogenously applied via sprays, submersion, painting, and coating (**Figure 1**). Peptide hormones regulate key agronomic and horticultural traits that significantly influencing the postharvest quality of fruits and vegetables (Ji et al., 2025; Zhang et al., 2025). In this regard, beyond exogenous application, gene editing or genetic transformation offers a promising avenue to modulate endogenous peptide levels, thereby harnessing their innate antimicrobial, antioxidative, and anti-aging properties to sustain quality and extend storage duration. Consequently, genetic improvement targeting functional peptide-encoding genes represents a fundamental strategy for addressing postharvest challenges in horticultural crops.

Importantly, peptide hormone-based composite materials can integrate multiple functional advantages to reduce postharvest losses. For instance, combining peptides with nanoparticles, polysaccharides, polyphenols, or nutrients can yield edible composites that simultaneously maintain quality and enhance nutritional value (Ranjith et al., 2022; Tkaczewska, 2020). Furthermore, peptide hormones can be synergistically combined with other preservation techniques, such as cold storage, modified atmosphere packaging, and edible coatings, to leverage complementary advantages and improve overall preservation efficacy.

In conclusion, despite significant challenges, ongoing technological advances and research efforts are poised to expand the application of peptide hormones in mitigating postharvest losses. By ensuring stability, safety, and cost-efficiency, peptide hormone-based strategies can contribute significantly to the sustainable development of the horticultural industry.

## References

[B1] Aghdam M. S. Alikhani-Koupaei M. (2021). Exogenous phytosulfokine α (PSKα) applying delays senescence and relief decay in strawberry fruits during cold storage by sufficient intracellular ATP and NADPH availability. Food Chem. 336, 127685., PMID: 32758803 10.1016/j.foodchem.2020.127685

[B2] Aghdam M. S. Alikhani-Koupaei M. Khademian R. (2021a). Delaying broccoli floret yellowing by phytosulfokine α application during cold storage. Front. Nutr. 8, 609217. 33869261 10.3389/fnut.2021.609217 PMC8047079

[B3] Aghdam M. S. Ebrahimi A. Sheikh-Assadi M. (2021b). Phytosulfokine α (PSKα) delays senescence and reinforces SUMO1/SUMO E3 ligase SIZ1 signaling pathway in cut rose flowers (*Rosa hybrida* cv. Angelina). Sci. Rep. 11, 23227., PMID: 34853400 10.1038/s41598-021-02712-2 PMC8636500

[B4] Aghdam M. S. Flores F. B. (2021). Employing phytosulfokine α (PSKα) for delaying broccoli florets yellowing during cold storage. Food Chem. 355, 129626. 33780792 10.1016/j.foodchem.2021.129626

[B5] Aghdam M. S. Flores F. B. Sedaghati B. (2021c). Exogenous phytosulfokine α (PSKα) application delays senescence and relieves decay in strawberry fruit during cold storage by triggering extracellular ATP signaling and improving ROS scavenging system activity. Sci. Hortic. 279, 109906.

[B6] Aghdam M. S. Sayyari M. Luo Z. (2021d). Exogenous phytosulfokine α application delays senescence and promotes antioxidant nutrient accumulation in strawberry fruit during cold storage by triggering endogenous phytosulfokine α signaling. Postharvest Biol. Tec. 175, 111473.

[B7] Aghdama M. S. Sayyari M. Luo Z. (2020). Exogenous application of phytosulfokine α (PSKα) delays yellowing and preserves nutritional quality of broccoli florets during cold storage. Food Chem. 333, 127481., PMID: 32663753 10.1016/j.foodchem.2020.127481

[B8] Ahmed T. M. K. Bakr M. M. Ahmed Q. A. (2022). Side effects of preservatives on human life. Sci. Res. Jr. Pharm. 2, 1–14.

[B9] Ali A. Tan Y. Medani K. Xia C. Abdullahi N. M. Mahmood I.. (2025). Horticultural postharvest loss’ and its socio-economic and environmental impacts. J. Environ. Manage. 373, 123458., PMID: 39616780 10.1016/j.jenvman.2024.123458

[B10] Bisson M. M. Kessenbrock M. Müller L. Hofmann A. Schmitz F. Cristescu S. M.. (2016). Peptides interfering with protein-protein interactions in the ethylene signaling pathway delay tomato fruit ripening. Sci. Rep. 6, 30634., PMID: 27477591 10.1038/srep30634 PMC4967898

[B11] Fan H. Liu H. Zhang Y. Zhang S. Liu T. Wang D.. (2022). Review on plant-derived bioactive peptides: biological activities, mechanism of action and utilizations in food development. J. Future Foods. 2, 143–159.

[B12] Fang H. Zuo J. Ma Q. Zhang X. Xu Y. Ding S.. (2024). Phytosulfokine promotes fruit ripening and quality via phosphorylation of transcription factor DREB2F in tomato. Plant Physiol. 194, 2739–2754., PMID: 38214105 10.1093/plphys/kiae012

[B13] Geylan G. Janet J. P. Tibo A. He J. Patronov A. Kabeshov M.. (2025). PepINVENT: generative peptide design beyond natural amino acids. Chem. Sci. 16, 8682–8696., PMID: 40248248 10.1039/d4sc07642g PMC12002334

[B14] Gomes B. A. F. Alexandre A. C. S. de Andrade G. A. V. Zabzini A. P. de Barros H. E. A. Silva L. M. S. F.. (2023). Recent advances in processing and preservation of minimally processed fruits and vegetables: A review-Part 2: Physical methods and global market outlook. Food Chem. Adv. 2, 100304.

[B15] Ji C. Li H. Zhang Z. Peng S. Liu J. Zhou Y.. (2025). The power of small signaling peptides in crop and horticultural plants. Crop J. 13, 656–667.

[B16] Krouk G. Szponarski W. Ruffel S. (2023). Unleashing the potential of peptides in agriculture and beyond. Trends Plant Sci. 28, 734–736., PMID: 37069001 10.1016/j.tplants.2023.03.025

[B17] Li A. Miao Y. Meng J. Niu L. Pan L. Lu Z.. (2022). Peptidome analysis of mesocarp in melting flesh and stony hard peach during fruit ripening. Sci. Agric. Sin. 55, 2202–2213.

[B18] Liang H. Zhu Y. Li Z. Jiang Y. Duan X. Jiang G.. (2025). Phytosulfokine treatment delays browning of litchi pericarps during storage at room temperature. Postharvest Biol. Tec. 219, 113262.

[B19] Liu Y. Hou Y. Yi B. Zhao Y. Bao Y. Wu Z.. (2024). Exogenous phytosulfokine α alleviates chilling injury of loquat fruit via regulating sugar, proline, polyamine and γ-aminobutyric acid metabolisms. Food Chem. 436, 137729., PMID: 37857197 10.1016/j.foodchem.2023.137729

[B20] Merino M. C. Guidarelli M. Negrini F. de Biase D. Pession A. Baraldi E.. (2019). Induced expression of the *Fragaria* × *ananassa Rapid alkalinization factor-33-like* gene decreases anthracnose ontogenic resistance of unripe strawberry fruit stages. Physiol. Mol. Plant Pathol. 20, 1252–1263., PMID: 31355517 10.1111/mpp.12837 PMC6715598

[B21] Milić D. Dick M. Mulnaes D. Pfleger C. Kinnen A. Gohlke H.. (2018). Recognition motif and mechanism of ripening inhibitory peptides in plant hormone receptor ETR1. Sci. Rep. 8, 3890., PMID: 29497085 10.1038/s41598-018-21952-3 PMC5832771

[B22] Ranjith F. H. Adhikari B. Muhialdin B. J. Yusof N. L. Mohammed N. K. Ariffin S. H.. (2022). Peptide-based edible coatings to control postharvest fungal spoilage of mango (*Mangifera indica* L.) fruit. Food Contr 135, 108789.

[B23] Song H. Wang X. Hu W. Yang X. Diao E. Shen T.. (2017). A cold-induced phytosulfokine peptide is related to the improvement of loquat fruit chilling tolerance. Food Chem. 232, 434–442., PMID: 28490095 10.1016/j.foodchem.2017.04.045

[B24] Sridhar A. Ponnuchamy M. Kumar P. S. Kapoor A.. (2021). Food preservation techniques and nanotechnology for increased shelf life of fruits, vegetables, beverages and spices: a review. Environ. Chem. Lett. 19, 1715–1735., PMID: 33192209 10.1007/s10311-020-01126-2 PMC7651826

[B25] Tadiello A. Ziosi V. Negri A. S. Noferini M. Fiori G. Busatto N.. (2016). On the role of ethylene, auxin and a GOLVEN-like peptide hormone in the regulation of peach ripening. BMC Plant Biol. 16, 44., PMID: 26863869 10.1186/s12870-016-0730-7 PMC4750175

[B26] Tkaczewska J. (2020). Peptides and protein hydrolysates as food preservatives and bioactive components of edible films and coatings-A review. Trends Food Sci. Technol. 106, 298–311.

[B27] Wan F. Kontogiorgos-Heintz D. de la Fuente-Nunez C. (2022). Deep generative models for peptide design. Digit Discov. 1, 195–208. 35769205 10.1039/d1dd00024a PMC9189861

[B28] Wang D. Huang H. Jiang Y. Duan X. Lin X. Aghdam M. S.. (2022). Exogenous phytosulfokine α (PSKα) alleviates chilling injury of banana by modulating metabolisms of nitric oxide, polyamine, proline, and γ-aminobutyric acid. Food Chem. 380, 132179., PMID: 35086014 10.1016/j.foodchem.2022.132179

[B29] Wang G. Zhang G. Wu M. (2016). CLE peptide signaling and crosstalk with phytohormones and environmental Stimuli. Front. Plant Sci. 6, 1211., PMID: 26779239 10.3389/fpls.2015.01211 PMC4703810

[B30] You Y. Zhou Y. Duan X. Mao X. Li Y.. (2022). Research progress on the application of different preservation methods for controlling fungi and toxins in fruit and vegetable. Crit. Rev. Food Sci. Nutr. 63, 12441–12452., PMID: 35866524 10.1080/10408398.2022.2101982

[B31] Zeng Y. Bao Y. Q. Shen X. Y. Liu Y. Yi B. H. Ren H. Y.. (2024). Phytosulfokine-α treatment maintains fruit quality and alleviates chilling injury of peach fruit by regulating relative oxygen species and cell wall metabolisms. Postharvest Biol. Tec. 216, 113073.

[B32] Zhang Z. Han H. Zhao J. Liu Z. Deng L. Wu L.. (2025). Peptide hormones in plants. Mol. Hortic. 5, 7. 39849641 10.1186/s43897-024-00134-y PMC11756074

[B33] Zhang Y. M. Ye D.-X. Liu Y. Zhang X.-Y. Zhou Y.-L. Zhang L.. (2023). Peptides, new tools for plant protection in eco-agriculture. Adv. Agrochem. 2, 58–78.

[B34] Zhao P. Yang H. Sun Y. Zhang J. Gao K. Wu J.. (2025). Targeted MYC2 stabilization confers citrus Huanglongbing resistance. Science 388, 191–198. 40208996 10.1126/science.adq7203

